# Application of smart phone in "Better Border Healthcare Program": A module for mother and child care

**DOI:** 10.1186/1472-6947-10-69

**Published:** 2010-11-03

**Authors:** Jaranit Kaewkungwal, Pratap Singhasivanon, Amnat Khamsiriwatchara, Surasak Sawang, Pongthep Meankaew, Apisit Wechsart

**Affiliations:** 1 Center of Excellence for Biomedical and Public Health Informatics (BIOPHICS) Faculty of Tropical Medicine, Mahidol University, Bangkok, Thailand; 2 Department of Tropical Hygiene, Faculty of Tropical Medicine, Rajavithi Campus, Mahidol University, Bangkok, Thailand; 3 Tanowsri Health Center, Suan Phung, Ratchaburi Provincial Health Office. Ratchaburi, Thailand

## Abstract

**Background:**

To assess the application of cell phone integrating into the healthcare system to improve antenatal care (ANC) and expanded programme on immunization (EPI) services for the under-served population in border area.

**Methods:**

A module combining web-based and mobile technology was developed to generate ANC/EPI visit schedule dates in which the healthcare personnel can cross-check, identify and update the mother's ANC and child's EPI status at the healthcare facility or at the household location when performing home visit; with additional feature of sending appointment reminder directly to the scheduled mother in the community.

**Results:**

The module improved ANC/EPI coverage in the study area along the country border including for both Thai and non-Thai mothers and children who were either permanent resident or migrants; numbers of ANC and EPI visit on-time as per schedule significantly increased; there was less delay of antenatal visits and immunizations.

**Conclusions:**

The module integrated and functioned successfully as part of the healthcare system; it is proved for its feasibility and the extent to which community healthcare personnel in the low resource setting could efficiently utilize it to perform their duties.

## Background

Even though the structure of healthcare system is well organized and distributed throughout Thailand, the system still does not function efficiently in many areas, especially in rural and remote communities. The difficulties in those under-served areas include not only the poverty of the communities but also the limited availability and access to healthcare services within the communities; both inhibit the treatment-seeking behaviors of the villagers. The project *Application of Smart Phone in "Better Border Healthcare Program" *was thus proposed and it was awarded by the Microsoft Research in early 2008. The main objective of this 2-year project was to develop technology-based healthcare solutions that will increase the accessibility and affordability of healthcare services.

The public health services proposed in the better border healthcare project were corresponding to the goals, in part, of the United Nations - Millennium Development Goals as well as to the public health key indicators of Thailand Ministry of Public Health. Three major goals of the Millennium Development Goals targeting at healthcare services include: (1) improve maternal health, (2) reduce child mortality, and (3) combat HIV/AIDS, malaria and other disease. In contrast to metropolitan areas and large cities/towns, the access to care in the rural areas remains a problem due to the limited health resources, lack of health concerns, and poor education. Surprisingly, the use of communication technology, i.e., cellular phone, is becoming popular outreaching even to those living in the remote areas. Thus, application of such technology might be a proven solution to alter treatment seeking behavior and create opportunities for better healthcare access.

The specific objective of the better border health project was therefore to develop models for utilizing smart cell phone as health communication tool: (1) to improve maternal health focusing on antenatal care (ANC), (2) to reduce child mortality focusing on the Expanded Programme on Immunization (EPI), and (3) to prevent/monitor disease incidence and to ensure treatment outcomes focusing on malaria due to malaria endemic in the border areas. The project consisted of 2 models: (1) Mother and Child Care Module (MCCM) and (2) Disease and Treatment Monitoring Module. This study focused on the results of MCCM implementation in the study area; the second module was described elsewhere.

The optimal number of ANC visits as well as the activities at the ANC visits have been discussed in literature [1-4]. The World Health Organization (WHO) recommends a typical ANC program for the minimum of four ANC visits for low risk pregnancies and prescribes certain activities at each visit [1,2]. Clinical activities usually include, for example, blood pressure measurement, urine testing for bacteriuria and proteinuria, blood testing to detect syphilis and severe anemia, and weight/height measurement (optional). Thailand Ministry of Public Health has recommended that the first antenatal consultation should be performed at the gestation age of approximately 6-8 weeks [5]. The practices in Thailand at all healthcare levels also follow ANC standard such that the healthcare provider would set extra appointments for re-examinations if any abnormality is found [6,7].

The EPI is a program launched in 1974 by the WHO in attempt to vaccinate and protect as many individuals as possible from preventable diseases including mainly six common diseases: polio, diphtheria, tuberculosis, pertussis, measles and tetanus [8,9]. Thailand had set its goal within the Universal Child Immunization Programme to fully immunize at least 80 percent of children under one year of age with one dose of Bacillus Calmette Guerin (BCG), three doses of Diphtheria, Pertussis and Tetanus (DTP), three doses of Oral Polio Vaccine (OPV) and one dose of Measles containing vaccine (MCV); all of these routine EPI vaccines are supported by the government [10,5]

The MCCM was developed under software development life cycle approach to cover the ANC/EPI activities mentioned above. The module has been integrating into the existing open-source Health Care Information System (HCIS) which is used by health centers across country. The main objective of this study was thus to assess the module effectiveness in improving antenatal care (ANC) and expanded programme on immunization (EPI) services for the under-served population in border area.

## Methods

### Setting and study population

The MCCM was implemented as a pilot project in one of the Thai-Myanmar border area, Suan Phung District. The area consists of seven sub-districts with one 30-bed community hospital and 13 health centers. Particularly, the target population comprises of 4200 villagers of all ages living in 7 hamlets in a total of 482 households in Tanowsri Sub-district. Tanowsri borders Myanmar on the western front and approximately 90% of its residents are ethnic Karen, many of which are holding designated *"Highland Area" *and *"Displaced Person" *identification cards and speak the ethnic language [11]. Most of residents are farmers and laborers, and live in small houses or bamboo shelters with thatched roofs. Even though the lack of Thai citizenship status does not prevent the ethnic group from access to treatment and care at low or no cost, but it is considered as not being part of the coverage under the national universal health care scheme, and thus health-related statistics of these individuals are usually under-reported [12].

Within the area, under Thailand Ministry of Public Health structure, there is one local healthcare clinic with one physician providing primary care service working in conjunction with unpaid 30 village health volunteers living in the villages. In 2007, based on data extraction and calculation from the healthcare clinic at the pilot study area (unpublished data), the statistics of maternal and child care were as follow: (1) Maternal ANC - There were 114 pregnant women and about 20% had less than 4 ANC visits. Of those, about 12 had adverse pregnancy outcomes including 3 abortion/miscarriage, 8 low birth weight, and 1 stillbirth. (2) Child EPI - The coverage included 110 children, and about 10% reported as having missed EPI scheduled appointments.

### MCCM module

Within the management of healthcare facilities under Thailand Ministry of Public Health, each healthcare clinic uses standardized open-source information system, so-called HCIS, in collecting primary healthcare data in its responsible areas. One of the standard data tables of the HCIS database contains ANC/EPI data. The data extracted from this ANC/EPI data table are used as baseline data elements of the MCCM; that means, the MCCM is an added-on function to the existing healthcare database system.

**Figure 1:**
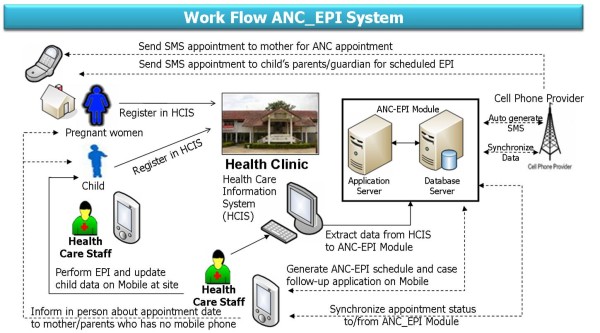
**Conceptual framework and work flow of MCCM**.

Two key functionalities of the MCCM include: (1) automated generation of listing and message reminder of mother and child with ANC/EPI visit due dates, and (2) update information regarding the antenatal care and child's immunization status on mobile phone when performing ANC/EPI activities off healthcare clinic. This module assists healthcare personnel in identifying case location and cross-checking personal history of ANC/EPI status while working in remote areas. Originally, the healthcare staff has to use a log book or printout from HCIS in managing ANC/EPI case follow-up at the healthcare clinic or within their responsible villages when performing routine home visits. Replacing the standard manual paper-based method with the mobile technology-based feature of the MCCM would make it more convenient and timely for healthcare personnel in monitoring ANC/EPI coverage and automatically updating the ANC/EPI status collected on the smart phone onto the HCIS database. Conceptual framework and work/data flow of the MCCM is summarized as shown in Figure 1.

**Figure 2:**
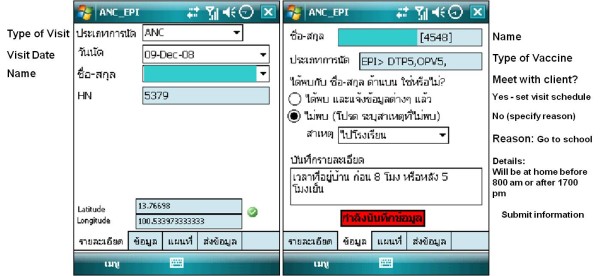
**Screen shots of appointment schedule and update visit status**.

The information regarding maternal care and child's immunization from the existing HCIS gets transmitted to the MCCM module. The program then generates appointment dates for each client and sends short message service (SMS) to healthcare worker for visit follow-up. If the mother or family members have listed phone numbers and allow the healthcare personnel to call them, the SMS will be automatically sent to them directly onto their own personal cell phone a few days prior to the scheduled date. For the clients without telephone, the healthcare staff will perform home visit as per scheduled reminder of visit dates and type of immunization shown on their smart cell phones. The visit status of the mother or child can then be updated onto the smart phone and synchronized collected data directly onto the system (see Figure 2).

The MCCM can also capture ANC/EPI visit locations of the mother and child in the area when healthcare staffs perform their routine visits as per standard practice of the local healthcare system in rural or remote area. This feature makes it easier for healthcare staff by not having to manually draw client's mapping for keep in the paper-based family folder which is the standard practice prior to the MCCM.

**Figure 3:**
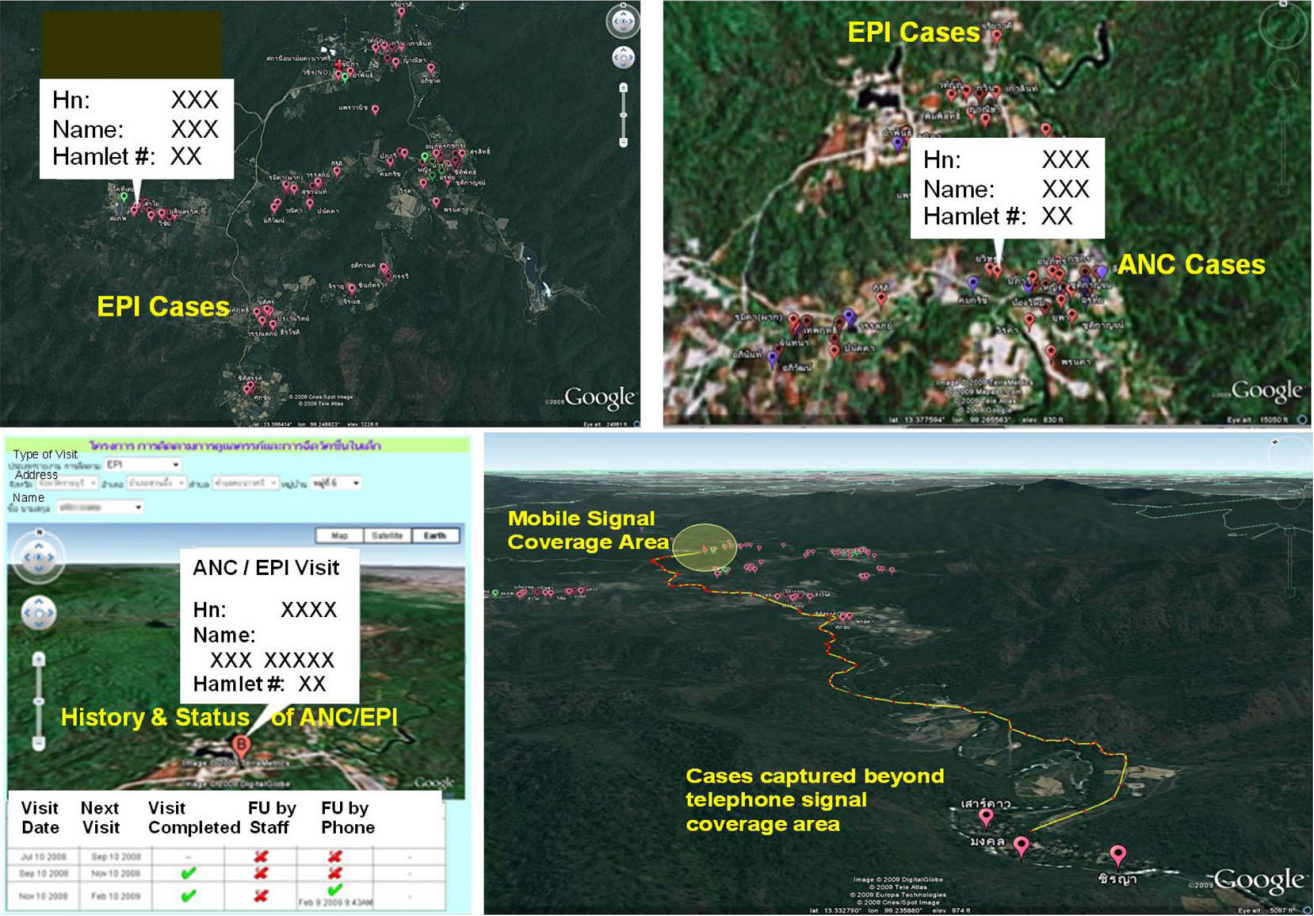
**Screen shots of ANC/EPI visits (case history & location map)**.

With the new functionality of visit schedule reminder, the healthcare staff can update the information regarding visit and activity completion at remote area if the ANC/EPI activities are not performed within the healthcare facility. The information captured on the phone can be done in the area where there is telephone signal coverage, or the area beyond the signal coverage and the healthcare staff can then synchronized information onto the system when signal is available. The mapping of all cases coverage within the health service areas could be seen at healthcare clinic as well as at the upper supervisory level. The history of ANC/EPI visits for each case could also be monitored as report or displayed on the location map (see examples of case mapping in Figure 3). The databases are strictly accessed and used by only authorized personnel in accordance with confidential and ethical consideration.

### Implementation and assessment of MCCM

The MCCM was implemented at the pilot testing area in December 2008 and was full-functioning since January 2009. The healthcare personnel who were in charge of ANC/EPI activities were equipped with smart phones that can handle text messages as well as data capturing in remote areas. They were trained to understand and apply as part of their routine works the new MCCM features and activities in place of the original paper-based activities.

The assessment of the MCCM application was planned to compare ANC/EPI data before and after its implementation. The antenatal care and child's immunization data were extracted from the standard data tables in the HCIS database of the healthcare clinic at pilot testing site, The data were also captured from the added-on data tables of the integrated MCCM and HCIS containing data about short message reminder transmission, and maternal and child care status updated on the healthcare provider's smart phone.

An indicator that could reflect the success of the MCCM application and utility is the compliance and punctuality to the scheduled ANC/EPI visits. In standard practice prior to MCCM, after each ANC visit the consecutive appointment date will be scheduled from the current visit date by the healthcare staff; in contrast, the MCCM automatically calculates each appointment date and transmits message reminder to the responsible healthcare provider as well as to the pregnant woman who is registered at the healthcare clinic. On the other hand for the EPI visit, it was originally managed by paper-based system in making visit schedule and vaccination plan. The healthcare personnel usually perform vaccinations and baby health care activities at a meeting location within each village/hamlet on a monthly preset date for all children who are scheduled for their immunizations around that date. With the MCCM, scheduled visit date and vaccine plan for each registered new born or baby residing in the village will be automatically generated and sent directly to the parents who have phone listed in the database. In this study, the punctuality of ANC/EPI visit was defined as the pregnant women coming to healthcare clinic for their ANC activities or the children coming to get immunization according to their scheduled vaccination plan on the predetermined appointment date. Specifically, the ANC attendance on-time was defined as the woman coming to each consecutive appointment date within a window period for ± 7 days. The EPI attendance on-time referred to the children coming on the appointment date.

As the ANC/EPI data of this study were collected from activities in natural settings of the healthcare facility in the pilot project areas, the analyses were thus based on actual cases and their scheduled visits before and after the MCCM implementation. There were pregnant women and children whose scheduled visits were in overlap period. The exploration of factors associated with being compliance and on-time with ANC/EPI visit schedule was thus based on visits using the Generalized Estimation Equation (GEE) model.

### Ethical consideration

It should be noted that all activities in the ANC/EPI management and database containing information associated with such activities are strictly accessed and used by only authorized healthcare personnel who are in charge of case management. The electronic system developed and used as part of MCCM maintains all crucial features of data integrity and confidentiality the same way as it has been routinely done in the paper-based process at the local healthcare clinic or during routine site/home visits.

No written informed consent or assent form was signed by mothers and/or children who visited the healthcare clinics or when meeting with healthcare personnel during site/home visits as all of the activities were routine work of the healthcare standard practices; however, the staff had verbally informed and asked the mothers/children to return to clinic or allow home visits as part of the scheduled follow-ups. The data extracted from the databases of HCIS and MCCM were secondary data with no identification-linked. The authors were granted for using the extracted data for analysis from the authorized person at the District Health Office. This study protocol was reviewed and approved by the Ethical Committee of the Faculty of Topical Medicine, Mahidol University.

## Results

### ANC visits

Data extracted from the HCIS database during 2007 to 2009 indicated that there had been consistent numbers of pregnancy and delivery; 123 and 100 women attended ANC clinic during 2008 and 2009 while 57 attended in between the two years. As shown in Table 1, about half of all women in the three cohorts were non-Thai (including Karen, Myanmar and Mon) with approximately 70% of no education. A few pregnant women aged 17 or less, mostly were in the age ranges of 20-30. The parity ranged from 1 to 4 or more similarly across the three cohorts; while most women had an average of 2-3 children, the maximum numbers of children were found up to 7-9 in about 5% across the groups.

**Table 1:** Characteristics of pregnant women who visited healthcare clinic for ANC

Characteristics	ANC during Year 2008 (n = 123)	ANC overlapping Year 2008-9 (n = 57)	ANC during Year 2009 (n = 100)
Nationality - n(%)			
Thai	70 (56.91)	31 (54.39)	55 (55.00)
Non-Thai	41 (33.33)	26 (45.61)	45 (45.00)
Missing	12 (9.76)	-	-
Education Level - n(%)			
No education	86 (69.92)	41 (71.93)	73 (73.00)
Primary education	18 (14.63)	13 (22.81)	15 (15.00)
Above Primary education	7 (5.69)	3 (5.26)	12 (12.00)
Missing	12 (9.76)	-	-
Age group - n(%)			
< 18 years	4 (3.25)	2 (3.51)	9 (9.00)
18-20 years	15 (12.20)	10 (17.54)	14 (14.00)
21-25 years	35 (28.46)	18 (31.58)	24 (24.00)
26-30 years	20 (16.26)	16 (28.07)	27 (27.00)
31-35 years	20 (16.26)	7 (12.28)	15 (15.00)
35-40 years	8 (6.50)	3 (5.26)	5 (5.00)
> 40 years	8 (6.50)	1 (1.75)	5 (5.00)
Missing	13 (10.57)	-	1 (1.00)
Parity - n(%)			
1	38 (31.15)	16 (28.07)	32 (32.00)
2	34 (27.87)	12 (21.05)	24 (24.00)
3	21 (17.21)	13 (22.81)	17 (17.00)
4 or more	29 (23.77)	16 (28.07)	27 (27.00)
Current Age			
Mean (SD)	27.28 (7.21)	25.60 (5.88)	26.24 (7.17)
Min-Max	16-46	16-45	14-47
Number of visits per ANC schedule*			
Mean (SD)	2.96 (1.22)	3.02 (1.14)	2.72 (1.22)
Min-Max	1-4	1-4	1-4
Number of actual ANC visits*			
Mean (SD)	2.83 (1.88)	4.00 (2.22)	3.23 (2.13)
Min-Max	1-8	1-11	1-10
Gestational age at ANC visits *			
Mean (SD)	23.48 (8.56)	19.21 (8.70)	20.63 (7.33)
Min-Max	8-42	8-40	7-39

* Many cases of ANC during 2009 are still on-going ANC visits in 2010

**Figure 4:**
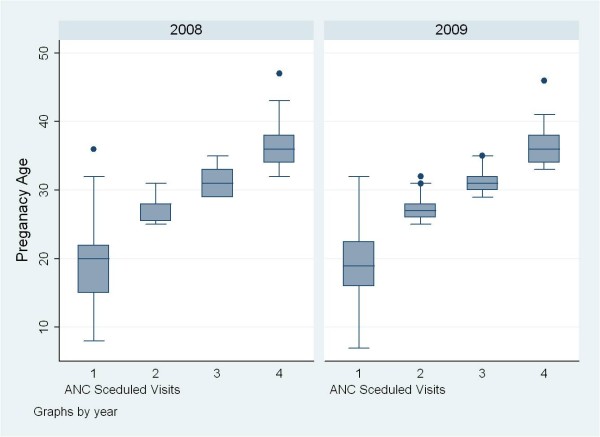
**Gestational age at ANC scheduled visits before and after MCCM**.

Despite the minimum of four ANC visits as per typical scheduled ANC appointment throughout the pregnancy period, average numbers of ANC visits were 2.96, 3.02 and 2.72 for women attending the healthcare clinic in 2008, 2008-2009 and 2009. The numbers of actual visits among the three cohorts ranged from 1-11 visits, the averages were 2.83 in cohort 2008, and then higher to 4.00 and 3.23 in cohorts 2008-9 and 2009 respectively. It should be taken into consideration that the number of ANC visits of the 2009 cohort has not yet finalized as several women are still on-going ANC visits in 2010. The average gestational ages of the three cohorts were shown somewhat different; 23 ± 9, 19 ± 9 and 21 ± 7 weeks respectively. However, when compared the gestational ages at the four recommended ANC schedule visits before and after MCCM implementation in 2009, no significantly difference were determined. The initial ANC visits in 2008 and 2009 was reported at the same gestational ages of approximately 18 weeks; and at the subsequent three schedule visits were about 27, 31 and 36 weeks (see Figure 4).

The 280 pregnant women attended 900 ANC visits during the study period; about 58.68% came on-time as per scheduled dates after MCCM implementation compared to 43.79% before the MCCM (p < 0.001); this increasing trend corresponded to the fact that about 10% of women actually received appointment message reminder on their cell phones and about 10% was updated ANC status by the healthcare personnel on their smart phones while performing home visits (table not shown). It should be noted that most women have usually come to health clinic by themselves and rather not missed ANC scheduled visits, but they tended not to come on-time as per schedule. Among the not on-time visits, the median was 14 days off-schedule and approximately 5% came about one month before scheduled date and 10% came about one month late (table not shown).

As shown in Table 2, after adjusted for personal characteristics of the mothers, sending appointment message on cell phone increased odds of visit on-time by 2.97 (1.60-5.54). The visit on-time was compared between year 2008 and 2009 and it was shown that the odds increased by 1.91 (1.46-2.49) after MCCM implementation.

**Table 2:** Factors associated with antenatal care visit on-time

Factors	OR (95%CI)*		Adjusted OR (95%CI)**	
Personal characteristics				
Nationality				
Thai	1			
Non-Thai	0.79	(0.60-1.04)		
Education				
No Education	1			
Primary Education	1.23	(0.85-1.75)		
Above Primary Education	1.12	(0.69-1.81)		
Age Group				
< 18 years	1			
18-20 years	0.70	(0.37-1.31)		
21-25 years	0.71	(0.40-1.28)		
26-30 years	0.51	(0.29-0.94)		
> 30 years	0.62	(0.35-1.11)		
Parity				
1	1			
2	1.00	(0.69-1.43)		
3	0.88	(0.60-1.28)		
4 or more	1.06	(0.74-1.51)		
Application of MCCM				
Follow-up cases on cell phone				
No	1		1	
Yes	1.04	(0.55-1.96)	0.99	(0.52-1.88)
Send appointment messages				
No	1		1	
Yes	3.07	(1.69-5.58)	2.97	(1.60-5.54)
Year				
2008 - Before MCCM implementation	1		1	
2009 - After MCCM implementation	1.83	(1.41-2.37)	1.91	(1.46-2.49)

* Odds Ratio (95% Confidence Interval) from Generalized Estimation Equation

** Adjusted for personal characteristics (nationality, education, age, parity)

### EPI visits

The MCCM-EPI module had followed cumulative 544 children, 396 cases before and 148 after the module implementation and actively functioned. As shown in Table 3, both sexes appear to be equally distributed; however, the higher percentage of Thai children was shown for EPI in 2009. The immunizations were administered according to the standard schedule set by Thailand Ministry of Public Health, ranging from at birth up to 6 years old with a few cases reported receiving EPI after 6 years old. For the children in the cohort before 2009, the median number of immunizations per child was 5 (IQR = 5); in contrast to the cohort of new cases in 2009, the median was 3 (IQR = 3). Of these 544 children, 2814 immunizations had been administered during mid 2007 to end of 2009 and almost all children are still under on-going EPI schedule. It should be noted, however, that these numbers might not reflect the actual numbers of immunizations the child had since there might be some missing, incomplete and non-reported incidences before the MCCM implementation.

**Table 3:** Characteristics of children who received EPI during 2008-2009

Characteristics	EPI cases before 2009 * (n = 396)	New EPI cases in 2009 ** (n = 148)
Gender		
Female	189 (47.73)	63 (42.57)
Male	205 (51.77)	85 (57.43)
Missing	2 (0.50)	-
Nationality - n(%)		
Thai	253 (63.89)	106 (71.62)
Non-Thai	141 (35.61)	41 (28.38)
Missing	2 (0.50)	-
Education - n(%)		
No education	390 (98.49)	138 (93.24)
Grade 1	4 (1.01)	7 (4.73)
Grade 2	-	3 (2.03)
Missing	2 (0.50)	-
Age group - n(%)		
< 1 year	-	65 (43.92)
1 year	61 (15.40)	46 (31.08)
2 year	110 (27.78)	6 (4.05)
3 year	118 (29.80)	3 (2.03)
4 year	67 (16.92)	8 (5.41)
5 year	33 (8.33)	1 (0.68)
6 year and above	5 (1.26)	19 (12.84)
Missing	2 (0.50)	-
Number of immunizations per child reported ***		
Median	5	3
25% - 75% Percentile	3-8	2-5
		

Immunizations received (n = 2814) ***	(n = 2266)	(n = 548)
BCG	30 (1.32)	34 (6.20)
DH	236 (10.41)	166 (30.29)
DTP	470 (20.74)	42 (7.66)
HBV	98 (4.32)	34 (6.20)
JE	604 (26.65)	36 (6.57)
Measles	170 (7.50)	33 (6.02)
OPV	658 (29.04)	203 (37.04)

* Data were extracted from cases receiving EPI from mid 2007-8; almost all cases are still on-going EPI visits

** Data were extracted from cases who were not reported as receiving EPI in 2007-8 but receiving in 2009; almost all cases are still on-going EPI visits.

*** There might be missing, incomplete or non-reported immunizations in earlier years before data extraction from the system.

After the MCCM-EPI was full-functioning, about 17% of the child's parents received appointment reminders on their cell phones, and about 45% of the child's immunization information was updated on the smart phones of the healthcare staff. There was 44.22% of children who came to receive scheduled vaccines on-time on the preset monthly immunization date after the MCCM implementation compared to 34.49% before the MCCM (p < 0.001); among the not on-time visits, almost all children received the scheduled vaccine(s) in the consecutive month (table not shown).

As shown in Table 4, after adjusted for personal characteristics of the children, follow-up cases and updating immunization data on cell phone increased odds of EPI on-time by 2.04 (1.66-2.52). Sending appointment message was also found increasing odds of receiving EPI on-time by 1.48 (1.09-2.03). The vaccination on-time was compared between years before and after MCCM-EPI appointment schedule was full-functioning; it was shown that the odds increased by 2.13 (1.79-2.52).

**Table 4:** Factors associated with child immunization visit on-time

Factors	OR (95%CI)*		Adjusted OR (95%CI)**	
Personal characteristics				
Gender				
Female	1			
Male	1.05	(0.86-1.29)		
Nationality				
Thai	1			
Non-Thai	0.88	(0.70-1.09)		
Age Group				
< 1 year	1			
1 year	1.80	(1.26-2.59)		
2 year	1.69	(1.19-2.41)		
3 year	1.39	(0.96-2.01)		
4 year	0.59	(0.36-0.96)		
5 year	0.05	(0.01-0.27)		
6 year or above	0.06	(0.01-0.49)		
Application of MCCM				
Follow-up cases on cell phone				
No	1		1	
Yes	1.80	(1.48-2.18)	2.04	(1.66-2.52)
Send appointment messages				
No	1		1	
Yes	1.37	(1.02-1.84)	1.48	(1.09-2.03)
Year				
2007-8 - Before visit schedule full-functioning	1		1	
2009 - After visit schedule full-functioning	1.84	(1.56-2.16)	2.13	(1.79-2.52)

* Odds Ratio (95% Confidence Interval) from Generalized Estimation Equation

** Adjusted for personal characteristics (gender, nationality, age)

## Discussion

### ANC and EPI coverage and practice

The ANC coverage in the study area included both Thai and non-Thai mothers who were either permanent residents or migrants. Most of the non-Thai in this area were Karen with a few Mon and Hill tribe. In a study along Thailand north-western border [13], the rates of deliveries by skilled attendants were higher in Mon and Shan areas (up north and beyond this study area) because women were able to travel to facilities across the border in Thailand; it was reported that Mon and Shan communities were also more likely to have more than four antenatal care visits, access to postnatal care at a hospital, and access to contraception than women from Karen communities. However, the majority of non-Thai in this study area had permanent address status and was treated according to the Thailand national standard.

The average number of ANC visits among pregnant women in this border area seems to increase reaching towards the four scheduled visits. The gestational age at initial ANC visit was 18 weeks in which following national guideline of recommended schedule visits. Thailand had set policy that the first antenatal consultation should be done prior to the 6th month of pregnancy; and as a standard practice, it is recommended that after the first ANC visit, there should be at least one visit each during month 6-7, month 7-8 and month 8-9, constituting a minimum of 4 visits to ensure effectiveness; otherwise, it would be considered as receiving inadequate antenatal care [14,15]. Thus, most of the pregnant women in the study area could be considered as having adequate antenatal care visits.

Personal characteristics explored in this study including ethnicity, age, education and parity of pregnant women were found not statistically associated with ANC attendance on-time. In contrast to a community-based case-control study conducted in Thailand [16] to characterize pregnant women in 120 villages who did not receive ANC over a 1-year period, it was found that 6.7% did not receive antenatal care. By univariate analysis, failure to receive antenatal care was found associated with some maternal factors including maternal age, parity, history of infant death and birth intervals; however, after multivariate analysis, only parity remained as significant factor.

In the WHO report, it appears that, at the national level, Thailand's child immunization effort has been successful for nearly two decades ago and the reporting coverage was almost 100 percent [17]. EPI has greatly reduced incidence of all the six diseases [17,18]. In this study, the required immunizations were distributed for all registered babies at healthcare clinic, both Thai and non-Thai. This might be due to the fact, in part, that the standard practice for EPI was administered by healthcare staff at village vicinity on the preset date. There have been studies in the region [13,19,20] that reported the factors affecting immunization status in limited infrastructure setting including household visits and/or mobile team utilizing an outreach site for immunization. Studies in other regions also confirmed the benefits of short distance to immunization site [21-24].

There have been several studies [13,19] in the region that reported the association between immunization status with socioeconomic characteristics, zone of residence and ethnic group but not with history of anti-neonatal care attendance. However, personal characteristics explored in this study including gender, ethnicity and age of the children were found not statistically associated with EPI attendance on-time.

### MCCM application and its effect

The study results have provided some evidence that the MCCM was, in part, effective in terms of improving the punctuality of ANC and EPI attendance. The MCCM-ANC appointment reminder sent directly to women's cell phones increased odds of visit on-time and cut off workload of healthcare staff in using paper-based for generating appointment as well as for case tracking in their responsible villages. Similar effect was shown for the MCCM-EPI for compliance to the vaccination schedule on-time of monthly appointment date. The MCCM-EPI also made it easier for healthcare staff in performing baby heath care follow-ups and updating immunization data on smart phone and cutting down paper-based activities.

Previous studies [19,21,25,26] had shown that direct communication was clearly effective in improving general primary health care as well as immunization coverage. A study in the region [19] reported that mothers' knowledge of the target diseases of immunization, schedule for immunization and number of times to visit the immunization site would significantly increased chances of having fully immunized children. Studies in other region [27] suggested that strategy of outreach services could provided successful health outcomes; for example, the Reaching Every District (RED) strategy that has been implemented in African countries by WHO and UNICEF since 2002 to improve stagnated routine immunization coverage showed good results due to outreach services.

This study revealed effective use of mobile technology, particularly the use of mobile phone for data collection and short message distribution, as a tool for outreaching health care services, particularly ANC and EPI coverage. Similar idea of employing text messages for healthcare providers to send reminders to patients had shown success in increasing patients' adherence in vaccination services, asthma care, diabetes control, outpatient clinic attendance in a tertiary hospital and primary health care [28-36]. The findings of this study and others suggested effective application of mobile technology in deliveries of healthcare services; this is due to the fact that mobile telephones increasingly penetrate into not only urban but also rural communities as they are low-cost, easily available and commonly used in everyday life.

### Limitations of the study

As the ANC/EPI data were extracted from the data tables in the HCIS database at the healthcare clinic in pilot module testing site over the years before and after the MCCM implementation, the study could be considered as a before-after design without control group, Though the before-after study design has advantage in its ability to measure changes or to assess impact of the intervention within the same population, it has certain disadvantages which may or may not occur at individual or collective level in every study. The prevalence of disadvantage(s) in the study depends upon the nature of intervention, the study population and the method of data collection. Even though the study population was rather static and there was no known counterpart program regarding maternal and child healthcare within the study area during the pilot study period, the changes observed in this study may partially due to unobserved extraneous factors. Despite the increases in punctuality rates for ANC/EPI attendance after the MCCM implementation have made it a promising tool for planning maternal and child healthcare program, it is possible that the rates observed in this study especially after the MCCM may be overestimated. Although the MCCM activities attempted to mimic the routine maternal and child healthcare program situation, efforts to conduct home visits and/or make follow-up appointment by healthcare staff may have been more diligent than normal practices in case management due to sensitizing with the program and equipment. Nevertheless, this finding may reflect what maternal and child healthcare management could have achieved in terms of promoting adherence to healthcare program and case monitoring in ideal situation in which the healthcare staff put full effort in performing their ANC/EPI activities.

## Conclusion

### MCCM usage

The MCCM has been successfully integrated into ANC/EPI operations at the rural and remote areas. The modified part does not alter the routine work of healthcare staff in performing their ANC/EPI duties but rather change the paper-based to be electronic-based for data capture and data management with an additional feature of mobile technology for data transfer process. The module makes it easier for healthcare staff in case follow-up for treatment and care. From the feedback via conversations with the healthcare staff that used the MCCM at the pilot study site, they expressed satisfaction with the system and requested for expansion of the module for other disease coverage. The results of this study confirm the notion that the success of any health information technology is due to its functionalities that could enable a dramatic transformation in the delivery of health care, making it safer, more effective, and more efficient [37].

### Module operation & management costs

In quantifying the costs of designing, implementing and maintenance of this module, there are certain factors required to taking into account. The estimates here are based on only hardware and software, not the personnel costs. As the MCCM is simply an added-on function to the existing HCIS which is implemented and absorbed by the Thailand Ministry of Public Health budgeting; the costs for this HCIS part include one computer and its maintenance. The HCIS is an open-source program developed by Thailand Ministry of Public Health and delivered to each healthcare clinic for its own installation and/or adaptation specifically to fit its own needs, if any. The MCCM has been developed by BIOPHICS with supported from the Microsoft Research Grant and its concept and programming parts are posted as open-source. The MCCM requires a server to manage data processing (appointment scheduling and visit updating) and text message delivery. Currently the server is located at BIOPHICS, but this can be put as part of the existing computer at healthcare clinic. The cost of SMS in Thailand is rather low, about 0.03 US $ per message. The low-cost smart phone that should be distributed for healthcare staff use to perform their duties is dropping down tremendously over the year; in 2009, the phone that used in MCCM was about 200 US $. The module works effectively under telephone signal coverage area and full internet access, but the activities and data updating can be done in areas where there is no signal. Since the healthcare clinic in the study area has already used the internet for other purposes, this cost has thus already been absorbed into the healthcare facility management. The investment for internet access in the remote area where there is not yet available would vary from one location to another.

### Challenges

The limitation in application of the current version of MCCM is that its functionalities are based on the use of data, in part, of the existing HCIS of Thailand Ministry of Public Health. This problem could be resolved by making the MCCM to be independent and interoperable modular for any healthcare systems such that it could be linked and adaptable to different settings and data structures. That means, back to the concept of system development life cycle, this would require enhancement and redesign some parts of the current module.

Key challenges in application of MCCM also lie in driving the existing best practices in healthcare with additional effective technology. One of the key issues is to make system users see the usefulness of the information they collect. In managing even the mandate HCIS, some healthcare staffs still consider it as a burden to do all data entering and processing. The acceptance of any system rely not only the ease of use but the meaningful and useful of the information at the level of data generating. The acceptance will then lead to maintaining both high data quality and timeliness. The system employing electronic-based in place of paper-based, and with simple technology used in daily life, in part, would increase acceptance as it reduces workload while improves desirable health outcomes.

This study revealed an innovative community-based healthcare module that could enhance the health of mother and child in the rural communities, the future consideration is about how to sustain this effective functionality and expand to the larger scale. As suggested in a USAID report [38], even the best designed and carefully implemented communication interventions will deliver few results if not properly supported by authorities. Strengthening healthcare infrastructure and additional financial support are needed in certain locations for sustainability and implementation at a national level. Finally, important lesson learned in this study was that the healthcare service delivery system, no matter paper-based or electronic-based, could be functioning efficiently only by the full efforts of the dedicated local staff.

## Competing interests

The authors declare that they have no competing interests. However, the development of the MCCM was supported by the Microsoft Research Award.

## Authors' contributions

JK: Designed and planned for the study, drafted the first version of the paper, submitted paper and approved the final version.

PS: Assisted in designing and planning the study, writing the submitted paper and approved the final version.

AK: Designed application module and monitored progress of the module, assisted writing the submitted paper and approved the final version.

SS: Designed and programmed the application module, monitored and maintained the module implementation, extracted data for analysis, assisted in writing the submitted paper and approved the final version.

PM: Assisted in application module development monitored and maintained the IT systems used for module functioning, assisted in writing the submitted paper and approved the final version.

AW: Administered and monitored application module at the pilot study area, supervised other local staff in module implementation, assisted in writing the submitted paper and approved the final version.

## Pre-publication history

The pre-publication history for this paper can be accessed here:

http://www.biomedcentral.com/1472-6947/10/69/prepub
